# Crystal structure of *C*-2-benzo­thia­zole-*N*-methyl­nitrone

**DOI:** 10.1107/S2056989015013262

**Published:** 2015-07-17

**Authors:** Roman Doroschuk

**Affiliations:** a Department of Inorganic Chemistry, Taras Shevchenko National University of Kyiv, 64 Volodymyrska Str., 01033 Kyiv, Ukraine

**Keywords:** crystal structure, benzo­thia­zole, nitrone, S⋯O attractive inter­action

## Abstract

The mol­ecule of the title compound {systematic name: *N*-[(benzo­thia­zol-2-yl)methyl­idene]methyl­amine *N*-oxide}, C_9_H_8_N_2_OS, is close to planar [maximum deviation from the mean plane = 0.081 (2) Å], its conformation being stabilized by a strong intra­molecular attractive S⋯O inter­action [2.6977 (16) Å]. In the crystal, mol­ecules are linked into centrosymmetric dimers by pairs of weak C—H⋯O hydrogen bonds.

## Related literature   

For the 1,3-dipolar cyclo­addition reaction of nitro­nes, see: Tufariello (1984 ▸); Torssell (1988 ▸). For the properties of benzo­thia­zole derivatives, see: Bradshaw *et al.* (2002 ▸); Paramashivappa *et al.* (2003 ▸); Jimonet *et al.* (1999 ▸); Ul-Hasan *et al.* (2002 ▸); Şener *et al.* (2000 ▸); Mruthyunjayaswamy & Shanthaveerappa (2000 ▸); Arpaci *et al.* (2002 ▸). For work by our group on nitro­nes, see: Doroschuk *et al.* (2006 ▸); Raspertova *et al.* (2002 ▸); Petkova *et al.* (2001 ▸). For attractive S—O inter­actions, see: Mokhir *et al.* (1999 ▸). For N—O bond lengths in nitro­nes, see: Ruano *et al.* (2012 ▸). For van der Waals radii, see: Wells (1986 ▸). For the synthesis, see: Delpierre & Lamchen (1965 ▸).
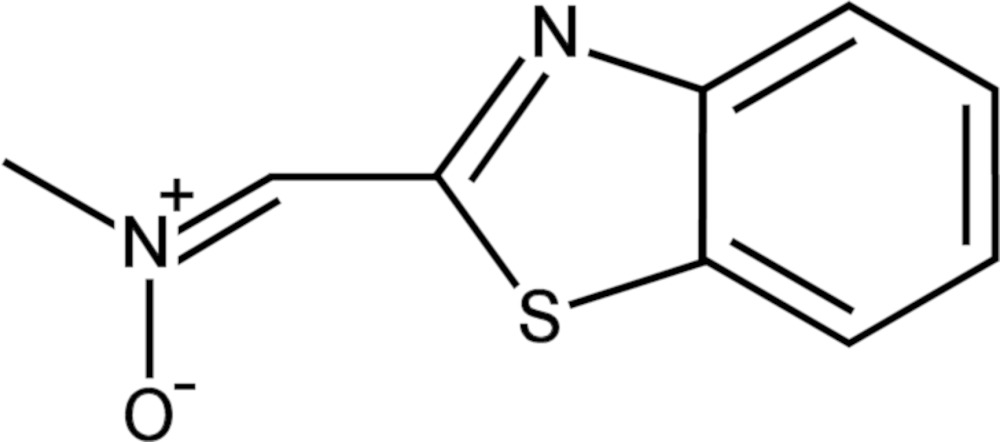


## Experimental   

### Crystal data  

C_9_H_8_N_2_OS
*M* *_r_* = 192.23
Triclinic, 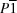

*a* = 5.5253 (14) Å
*b* = 7.4528 (19) Å
*c* = 10.839 (4) Å
α = 83.51 (2)°
β = 85.79 (3)°
γ = 77.39 (3)°
*V* = 432.2 (2) Å^3^
*Z* = 2
Mo *K*α radiation
μ = 0.33 mm^−1^
*T* = 294 K
0.4 × 0.3 × 0.2 mm

### Data collection  

Oxford Diffraction Xcalibur 3 diffractometer
Absorption correction: multi-scan (*CrysAlis PRO*; Agilent, 2011 ▸) *T*_min_ = 0.423, *T*_max_ = 0.994
3491 measured reflections
1933 independent reflections
1477 reflections with *I* > 2σ(*I*)
*R*_int_ = 0.018

### Refinement  

*R*[*F* ^2^ > 2σ(*F* ^2^)] = 0.033
*wR*(*F* ^2^) = 0.087
*S* = 1.00
1933 reflections
130 parameters
H atoms treated by a mixture of independent and constrained refinement
Δρ_max_ = 0.24 e Å^−3^
Δρ_min_ = −0.19 e Å^−3^

### 

Data collection: *CrysAlis PRO* (Agilent, 2011 ▸); cell refinement: *CrysAlis PRO*; data reduction: *CrysAlis PRO*; program(s) used to solve structure: *SHELXT* (Sheldrick, 2015*a* ▸); program(s) used to refine structure: *SHELXL2014* (Sheldrick, 2015*b* ▸); molecular graphics: *OLEX2* (Dolomanov *et al.*, 2009 ▸); software used to prepare material for publication: *OLEX2*.

## Supplementary Material

Crystal structure: contains datablock(s) I. DOI: 10.1107/S2056989015013262/rz5162sup1.cif

Structure factors: contains datablock(s) I. DOI: 10.1107/S2056989015013262/rz5162Isup2.hkl

Supporting information file. DOI: 10.1107/S2056989015013262/rz5162Isup3.cml

**Fig. 1.**
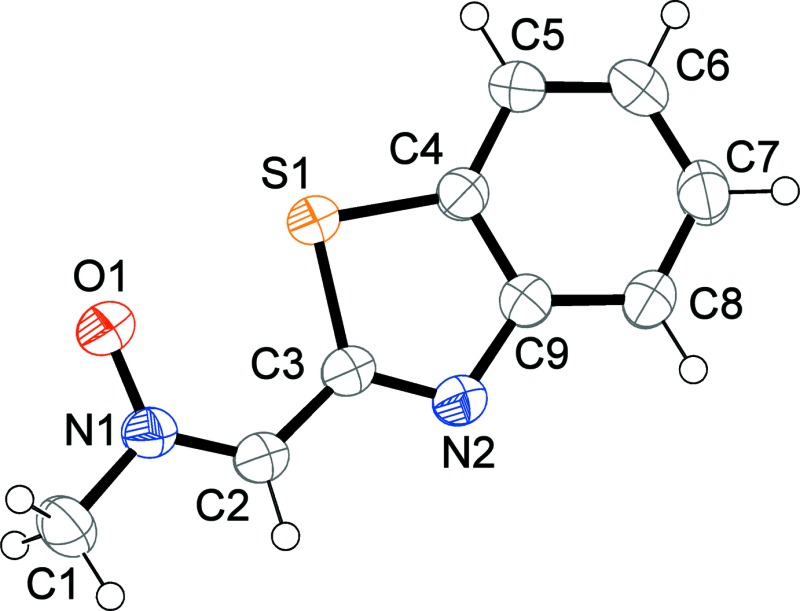
The molecular structure of the title compound with displacement ellipsoids drawn at the 50% probability level.

. DOI: 10.1107/S2056989015013262/rz5162fig1.tif
The mol­ecular structure of the title compound with displacement ellipsoids drawn at the 50% probability level.

**Fig. 2.**
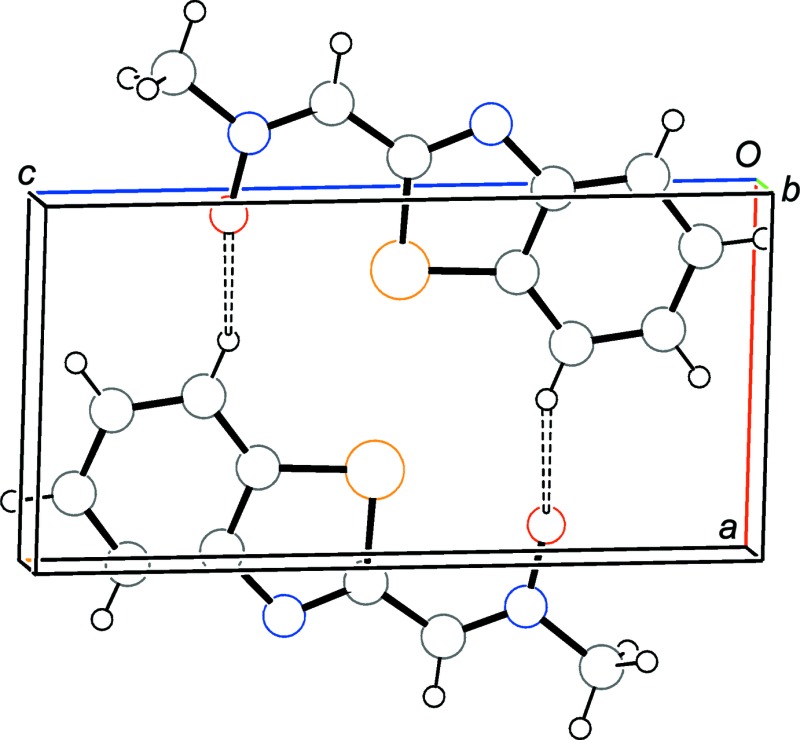
Partial crystal packing of the title compound approximately viewed along [0 1 0], showing the formation of a dimeric unit through a pair of C—H···O hydrogen bonds (dashed lines).

. DOI: 10.1107/S2056989015013262/rz5162fig2.tif
Partial crystal packing of the title compound approximately viewed along [0 1 0], showing the formation of a dimeric unit through a pair of C—H⋯O hydrogen bonds (dashed lines).

CCDC reference: 1411951

Additional supporting information:  crystallographic information; 3D view; checkCIF report

## Figures and Tables

**Table 1:** Hydrogen-bond geometry (, )

*D*H*A*	*D*H	H*A*	*D* *A*	*D*H*A*
C5H5O1^i^	0.93	2.53	3.331(2)	145

Symmetry code: (i) 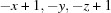.

## supplementary crystallographic information

### S1. Chemical context

One of the most important approaches for the symthesis of various five-membered heterocyclic systems is the 1,3-dipolar cyclo­addition reaction. The use as dipoles of compounds such as nitrile oxides and nitro­nes leads to a wide range of N,O-containing heterocyclic systems (isoxazole, isoxazoline, isoxazolidine; Tufariello, 1984), which have biological activity and can be used as starting materials for the synthesis of acyclic compounds (for example, 1,3-amino­alcohols by cleavage of the N–O bond; Torssell, 1988). On the other hand, compounds containing the benzo­thia­zole moiety are of biological and industrial inter­est. In fact, benzo­thia­zole derivatives possess a wide spectrum of biological applications such as anti­tumor (Bradshaw *et al.*, 2002), anti-inflammatory (Paramashivappa *et al.*, 2003), anti­convulsant (Jimonet *et al.*, 1999), and anti­microbial activities (Ul-Hasan *et al.*, 2002; Şener *et al.*, 2000; Mruthyunjayaswamy *et al.*, 2000; Arpaci *et al.*, 2002).

Following our studies on aromatic nitrone (Doroschuk *et al.*, 2006; Raspertova *et al.*, 2002; Petkova *et al.*, 2001) in this paper we describe the structure of *C*-2-benzo­thia­zole-*N*-methyl­nitrone.

### S2. Structural commentary

In the molecule of the title compound (Fig. 1), the oxygen atom of the nitrone group exists in *syn*-conformation with respect to the sulfur atom of the benzo­thia­zole moiety. This conformation is achieved due to a strong electrostatic intra­molecular attractive S···O inter­action. Thus, the S1···O1 distance (2.6977 (16) Å) is significantly shorter than the sum of the van der Waals radii of O and S (1.5 and 1.85 Å, respectively; Wells, 1986). A similar attractive S···O inter­action is characteristic for molecules containing the thia­zole moiety (Mokhir *et al.*, 2002). The value of the N1—C2 bond length (1.298 (2) Å) is typical for a C═N bond (1.28 Å; Wells, 1986), while the N1—O1 bond length (1.2763 (17) Å) is slightly shorter than those usually observed for nitrone N—O bonds (Petkova *et al.*, 2001; Ruano *et al.*, 2012). The heterocyclic ring system and the nitrone fragment C1—N1—O1—C2 are almost coplanar (the maximum deviation from the least-squares mean plane is 0.081 (2) Å for atom C1), forming a dihedral angle of 3.40 (9)°. The bond lengths within the benzo­thia­zole ring system are unexceptional.

### S3. Supra­molecular features

In the crystal (Fig. 2), centrosymmetrically-related molecules are linked into dimers *via* pairs of C—H···O hydrogen bonds (Table 1).

### S4. Synthesis and crystallization

*C*-2-Benzo­thia­zole-*N*-methyl­nitrone was synthesized by condensation of the corresponding aldehyde with N-methyl­hydroxyl­amine hydro­chloride in the presence of base (Delpierre & Lamchen, 1965).

2-Benzo­thia­zolecarbaldehyde (0.1632 g, 0.01 mol), CH_2_CL_2_ (20 ml), and *N*-metyl­hydroxyl­amine hydro­chloride (0.9175 g, 0.011 mol) were placed in a 50 mL flask, and the mixture was stirred at room temperature. To the resulting solution was added NaHCO_3_ (2.7675 g, 0.033 mol). The reaction mixture was reluxed for 3 h. The NaCl precipitate and excess of sodium bicarbonate was removed by filtration, and the filtrate was evaporated to give a solid (1.83g, yield 95%). The solid was recrystallized from an absolute hexane solution.

### S5. Refinement

One aromatic H atom (H6) could be located in a difference Fourier map and was refined freely. All other H atoms were placed geometrically and treated as riding, with C—H = 0.93-0.96 Å, and *U*_iso_(H) = 1.5*U*_eq_(C) for methyl H atoms or 1.2*U*_eq_(C) otherwise. A rotating model was used for the methyl H atoms. 36 outliers were omitted in the last cycles of refinement.

### Crystal data

**Table:**

C_9_H_8_N_2_OS	*Z* = 2
*M**_r_* = 192.23	*F*(000) = 200
Triclinic, *P*1	*D*_x_ = 1.477 Mg m^−^^3^
*a* = 5.5253 (14) Å	Mo *K*α radiation, λ = 0.71073 Å
*b* = 7.4528 (19) Å	Cell parameters from 110 reflections
*c* = 10.839 (4) Å	θ = 18.2–8.2°
α = 83.51 (2)°	µ = 0.33 mm^−^^1^
β = 85.79 (3)°	*T* = 294 K
γ = 77.39 (3)°	Block, colourless
*V* = 432.2 (2) Å^3^	0.4 × 0.3 × 0.2 mm

### Data collection

**Table:**

Oxford Diffraction Xcalibur 3 diffractometer	1477 reflections with *I* > 2σ(*I*)
Detector resolution: 16.1827 pixels mm^-1^	*R*_int_ = 0.018
ω scans	θ_max_ = 27.5°, θ_min_ = 2.8°
Absorption correction: multi-scan (*CrysAlis PRO*; Agilent, 2011)	*h* = −7→7
*T*_min_ = 0.423, *T*_max_ = 0.994	*k* = −9→9
3491 measured reflections	*l* = −14→14
1933 independent reflections	

### Refinement

**Table:**

Refinement on *F*^2^	Primary atom site location: structure-invariant direct methods
Least-squares matrix: full	Hydrogen site location: inferred from neighbouring sites
*R*[*F*^2^ > 2σ(*F*^2^)] = 0.033	H atoms treated by a mixture of independent and constrained refinement
*wR*(*F*^2^) = 0.087	*w* = 1/[σ^2^(*F*_o_^2^) + (0.0521*P*)^2^] where *P* = (*F*_o_^2^ + 2*F*_c_^2^)/3
*S* = 1.00	(Δ/σ)_max_ < 0.001
1933 reflections	Δρ_max_ = 0.24 e Å^−^^3^
130 parameters	Δρ_min_ = −0.19 e Å^−^^3^
0 restraints	

### Special details

**Table:**

Geometry. All e.s.d.'s (except the e.s.d. in the dihedral angle between two l.s. planes) are estimated using the full covariance matrix. The cell e.s.d.'s are taken into account individually in the estimation of e.s.d.'s in distances, angles and torsion angles; correlations between e.s.d.'s in cell parameters are only used when they are defined by crystal symmetry. An approximate (isotropic) treatment of cell e.s.d.'s is used for estimating e.s.d.'s involving l.s. planes.

### Fractional atomic coordinates and isotropic or equivalent isotropic displacement parameters (Å^2^)

**Table:**

	*x*	*y*	*z*	*U*_iso_*/*U*_eq_	
O1	0.0619 (2)	0.21490 (18)	0.72954 (11)	0.0573 (3)	
S1	0.22145 (7)	0.17869 (5)	0.49058 (4)	0.04032 (14)	
N1	−0.1621 (2)	0.29247 (17)	0.70659 (12)	0.0406 (3)	
N2	−0.1774 (2)	0.33921 (18)	0.37522 (12)	0.0415 (3)	
C1	−0.3325 (3)	0.3346 (3)	0.81411 (16)	0.0536 (4)	
H1A	−0.2936	0.4351	0.8513	0.091 (8)*	
H1B	−0.3159	0.2277	0.8738	0.078 (7)*	
H1C	−0.5000	0.3688	0.7878	0.100 (8)*	
C2	−0.2414 (3)	0.3343 (2)	0.59501 (14)	0.0402 (3)	
H2	−0.4066	0.3938	0.5861	0.048 (5)*	
C3	−0.0891 (3)	0.2941 (2)	0.48533 (14)	0.0366 (3)	
C4	0.2333 (3)	0.1897 (2)	0.33030 (14)	0.0368 (3)	
C5	0.4328 (3)	0.1236 (2)	0.25071 (15)	0.0423 (4)	
H5	0.5858	0.0644	0.2813	0.049 (5)*	
C6	0.3978 (3)	0.1484 (2)	0.12546 (16)	0.0470 (4)	
H6	0.531 (3)	0.098 (2)	0.0729 (18)	0.058 (5)*	
C7	0.1702 (3)	0.2371 (2)	0.07882 (15)	0.0503 (4)	
H7	0.1516	0.2513	−0.0065	0.055 (5)*	
C8	−0.0273 (3)	0.3040 (2)	0.15698 (15)	0.0483 (4)	
H8	−0.1790	0.3640	0.1253	0.065 (6)*	
C9	0.0036 (3)	0.2802 (2)	0.28572 (14)	0.0385 (3)	

### Atomic displacement parameters (Å^2^)

**Table:**

	*U*^11^	*U*^22^	*U*^33^	*U*^12^	*U*^13^	*U*^23^
O1	0.0454 (7)	0.0711 (8)	0.0454 (7)	0.0102 (6)	−0.0084 (5)	−0.0037 (6)
S1	0.0344 (2)	0.0451 (2)	0.0377 (2)	0.00088 (15)	−0.00453 (15)	−0.00450 (15)
N1	0.0385 (7)	0.0394 (7)	0.0402 (7)	−0.0011 (5)	−0.0012 (5)	−0.0029 (5)
N2	0.0322 (7)	0.0500 (7)	0.0399 (7)	−0.0034 (6)	−0.0031 (5)	−0.0038 (6)
C1	0.0562 (11)	0.0594 (11)	0.0411 (9)	−0.0060 (8)	0.0077 (8)	−0.0069 (8)
C2	0.0344 (8)	0.0425 (8)	0.0415 (8)	−0.0033 (6)	−0.0033 (6)	−0.0032 (7)
C3	0.0331 (7)	0.0355 (8)	0.0404 (8)	−0.0051 (6)	−0.0043 (6)	−0.0030 (6)
C4	0.0367 (8)	0.0362 (7)	0.0375 (8)	−0.0072 (6)	−0.0035 (6)	−0.0035 (6)
C5	0.0374 (8)	0.0431 (8)	0.0443 (8)	−0.0024 (6)	−0.0012 (7)	−0.0082 (7)
C6	0.0472 (9)	0.0492 (9)	0.0443 (9)	−0.0083 (7)	0.0086 (7)	−0.0133 (7)
C7	0.0555 (10)	0.0605 (10)	0.0359 (8)	−0.0128 (8)	−0.0020 (7)	−0.0081 (8)
C8	0.0420 (9)	0.0608 (10)	0.0402 (9)	−0.0055 (7)	−0.0086 (7)	−0.0030 (8)
C9	0.0369 (8)	0.0410 (8)	0.0376 (8)	−0.0076 (6)	−0.0024 (6)	−0.0049 (6)

### Geometric parameters (Å, º)

**Table:**

O1—N1	1.2763 (17)	C2—C3	1.426 (2)
S1—C3	1.7456 (16)	C4—C5	1.386 (2)
S1—C4	1.7269 (17)	C4—C9	1.393 (2)
N1—C1	1.461 (2)	C5—H5	0.9300
N1—C2	1.298 (2)	C5—C6	1.371 (2)
N2—C3	1.303 (2)	C6—H6	0.934 (19)
N2—C9	1.377 (2)	C6—C7	1.389 (3)
C1—H1A	0.9600	C7—H7	0.9300
C1—H1B	0.9600	C7—C8	1.371 (2)
C1—H1C	0.9600	C8—H8	0.9300
C2—H2	0.9300	C8—C9	1.405 (2)
			
C4—S1—C3	88.33 (8)	C5—C4—C9	121.64 (14)
O1—N1—C1	116.50 (13)	C9—C4—S1	109.96 (12)
O1—N1—C2	123.49 (13)	C4—C5—H5	121.0
C2—N1—C1	120.01 (13)	C6—C5—C4	117.98 (15)
C3—N2—C9	109.93 (12)	C6—C5—H5	121.0
N1—C1—H1A	109.5	C5—C6—H6	117.3 (12)
N1—C1—H1B	109.5	C5—C6—C7	121.45 (15)
N1—C1—H1C	109.5	C7—C6—H6	121.2 (12)
H1A—C1—H1B	109.5	C6—C7—H7	119.6
H1A—C1—H1C	109.5	C8—C7—C6	120.85 (15)
H1B—C1—H1C	109.5	C8—C7—H7	119.6
N1—C2—H2	118.2	C7—C8—H8	120.6
N1—C2—C3	123.58 (14)	C7—C8—C9	118.84 (16)
C3—C2—H2	118.2	C9—C8—H8	120.6
N2—C3—S1	116.37 (11)	N2—C9—C4	115.40 (14)
N2—C3—C2	121.29 (13)	N2—C9—C8	125.36 (14)
C2—C3—S1	122.33 (11)	C4—C9—C8	119.24 (15)
C5—C4—S1	128.40 (12)		
			
O1—N1—C2—C3	1.7 (2)	C4—S1—C3—C2	178.11 (13)
S1—C4—C5—C6	179.91 (13)	C4—C5—C6—C7	0.1 (3)
S1—C4—C9—N2	−0.08 (17)	C5—C4—C9—N2	−179.70 (14)
S1—C4—C9—C8	−179.91 (12)	C5—C4—C9—C8	0.5 (2)
N1—C2—C3—S1	1.4 (2)	C5—C6—C7—C8	0.4 (3)
N1—C2—C3—N2	−179.90 (14)	C6—C7—C8—C9	−0.4 (3)
C1—N1—C2—C3	−178.46 (14)	C7—C8—C9—N2	−179.78 (15)
C3—S1—C4—C5	179.96 (15)	C7—C8—C9—C4	0.0 (2)
C3—S1—C4—C9	0.38 (11)	C9—N2—C3—S1	0.71 (17)
C3—N2—C9—C4	−0.39 (19)	C9—N2—C3—C2	−178.07 (13)
C3—N2—C9—C8	179.43 (15)	C9—C4—C5—C6	−0.5 (2)
C4—S1—C3—N2	−0.65 (12)		

### Hydrogen-bond geometry (Å, º)

**Table:**

*D*—H···*A*	*D*—H	H···*A*	*D*···*A*	*D*—H···*A*
C5—H5···O1^i^	0.93	2.53	3.331 (2)	145

Symmetry code: (i) −*x*+1, −*y*, −*z*+1.
