# Supplementary figures and images for: Crystal structure of C-2-benzo­thia­zole-N-methyl­nitrone
Source: Acta Crystallogr E Crystallogr Commun. 2015 Jul 17;71(Pt 8):o578–9. doi: 10.1107/S2056989015013262 (PMC4571406; doi:10.1107/S2056989015013262)

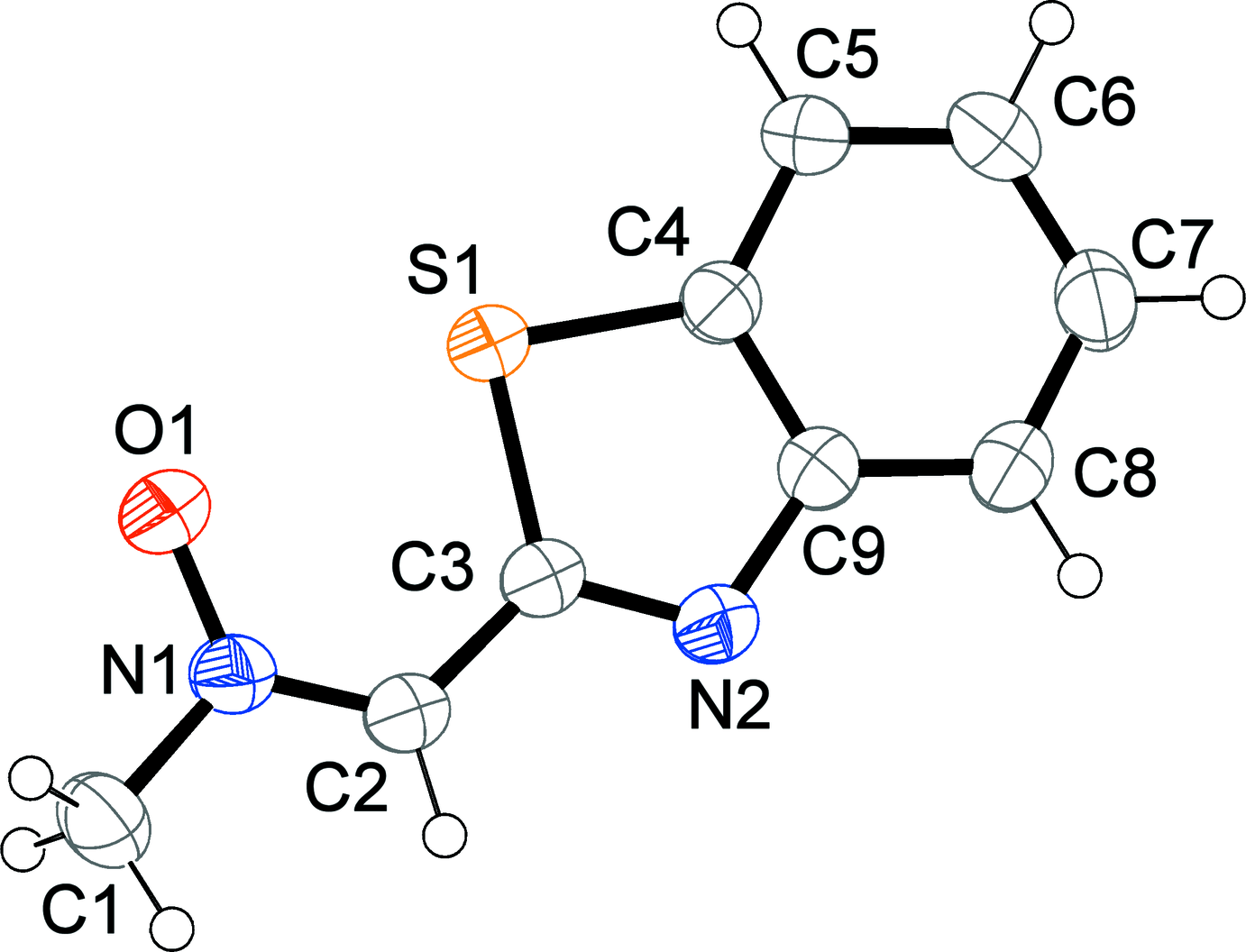

Supplement: Supplementary file 4 [file e-71-0o578-fig1.tif]

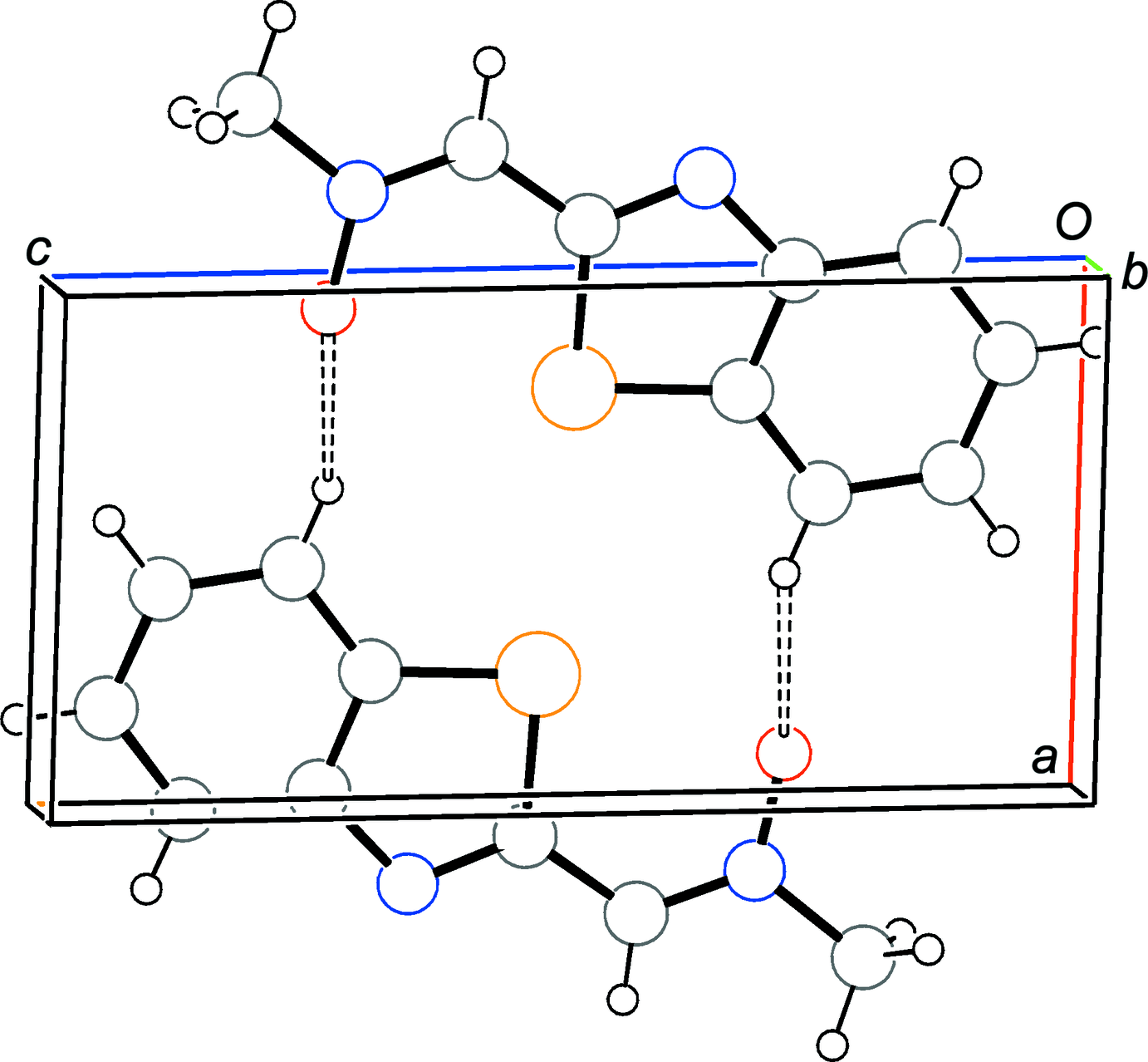

Supplement: Supplementary file 5 [file e-71-0o578-fig2.tif]
